# Statistical Optimization of Bacterial Cellulose Production and Its Application for Bacteriophage Immobilization

**DOI:** 10.3390/ijms26136059

**Published:** 2025-06-24

**Authors:** Grzegorz Skaradziński, Tomasz Janek, Paulina Śliwka, Aneta Skaradzińska, Wojciech Łaba

**Affiliations:** Department of Biotechnology and Food Microbiology, Faculty of Biotechnology and Food Science, Wrocław University of Environmental and Life Sciences, 50-375 Wrocław, Poland

**Keywords:** bacterial cellulose, phage, bacteriophage, carrier, optimization, fructose, carbon source

## Abstract

Bacterial cellulose (BC), an extracellular polysaccharide synthesized by various bacterial strains. It exhibits high tensile strength, water retention, crystallinity, and biocompatibility, making it valuable in biomedical, cosmetic, food, textile, and paper industries. This study examined the effects of six carbon sources on BC production by *Komagataeibacter sucrofermentans*, identifying fructose as the most effective. A Box–Behnken experimental design was employed to investigate the effects of three variables (fructose concentration, temperature, and cultivation time) on cellulose yield. The optimized cultivation conditions were: fructose concentration of 227.5 g/L, temperature of 28.0 °C, and cultivation time of 295 h, resulting in a BC yield of 63.07 ± 2.91 g/L. Subsequently, BC’s potential as a bacteriophage carrier was assessed. *Escherichia coli* phage T4 and *Staphylococcus aureus* phage vB_SauS_CS1 (CS1) were immobilized within BC hydrogels, and their antibacterial activities were assessed through in vitro experiments. These findings suggest BC’s promise as a phage delivery platform for biomedical applications.

## 1. Introduction

Bacterial cellulose (BC) is a natural extracellular polysaccharide, synthesized primarily by acetic acid bacteria such as *Komagataeibacter xylinus*. Structurally, BC consists of linear β-1,4-glucan chains that form a highly crystalline nanofibrillar network known as microfibrils [1]. In contrast to plant-derived cellulose, BC is free of lignin and hemicellulose, which contributes to its superior purity and physicochemical properties, including high tensile strength, excellent water retention, biocompatibility, and thermal stability [2]. Due to its safety profile, the U.S. Food and Drug Administration (FDA) classified BC as Generally Recognized as Safe (GRAS) in 1992 [3].

A variety of bacterial genera have been identified as BC producers, including species from *Komagataeibacter xylinus*, *Gluconacetobacter*, *Gluconobacter*, *Agrobacterium*, *Acetobacter*, *Rhizobium*, and *Pseudomonas* [4,5,6], as well as certain yeast species, such as *Pichia kudriavzevii* [7]. To meet the growing demand for BC across various applications, extensive efforts have been made to improve its production efficiency. Strategies aimed at increasing BC yield include strain selection, substrate optimization, and adjustment of cultivation parameters such as pH, temperature, and oxygen availability [8,9]. Notably, the physical properties of BC, such as porosity, density, and morphology, can vary significantly, depending on the cultivation method used, including static, agitated, or bioreactor-based systems [10].

To further tailor its mechanical and functional characteristics, BC can be modified either during biosynthesis by incorporating other polymers or post-synthetically, through the use of cross-linking agents [11]. Additionally, recent studies have reported enhanced BC productivity through co-culture strategies, such as combining *K. xylinus* with *Bacillus cereus* [12]. The substitution of conventional substrates like glucose with agricultural or industrial byproducts, such as fig waste or glycerol, has also been shown to reduce production costs, while maintaining or even improving BC yields [13].

Owing to its distinctive physicochemical properties, BC has been widely investigated for a wide range of applications. In industrial contexts, it serves as a reinforcing agent in recycled paper, a structural component in food packaging, and a dietary additive in low-fat and plant-based food formulations [14,15]. In the biomedical field, BC has found applications in wound care products, artificial dermal substitutes, dental membranes, vascular grafts, targeted drug delivery systems, and scaffolds for tissue engineering [16,17,18]. Furthermore, its role as a carrier matrix for probiotics and bioactive compounds highlights the material’s versatility in both biomedical and biotechnological applications [19]. Among its diverse biomedical applications, BC has recently garnered attention as a potential carrier for antimicrobial agents, including bacteriophages (phages), due to its excellent biocompatibility and structural properties [20,21].

Phages are viruses capable of infecting and lysing bacterial cells. They are extremely diverse in size, morphology, and genomic organization. However, all share a common structural feature—a nucleic acid genome encased in a protein capsid. Typically, bacteriophages exhibit a narrow host range, infecting only a specific bacterial species or even particular strains within that species [22]. Their antibacterial properties have been used to develop bacteriophage therapy, which relies on the use of phages to infect and lyse bacteria at the site of infection [23].

Phages can be delivered to infection sites using various formulations. Depending on the infection site (e.g., external or intracorporeal), several delivery strategies are available, including free phage administration, encapsulation in liposomes or alginate macrospheres, entrapment in hydrogels [24,25], and sponge-like chitosan wound dressings [26].

Given the multifunctional properties of BC as a biocompatible carrier, this study aimed to evaluate its potential as an innovative delivery platform for bacteriophages. BC was biosynthesized under previously optimized cultivation conditions using *K. sucrofermentans*. In the subsequent stage of the study, the ability of bacteriophages to be immobilized on BC was assessed, followed by an in vitro evaluation of the antibacterial properties of the immobilized phages. It is important to emphasize that existing literature offers very limited data regarding the potential application of BC as a carrier for bacteriophages. While BC has previously been employed as a platform for phage immobilization in *Staphylococcus aureus* detection systems, to the best of our knowledge, the present study is the first to demonstrate the feasibility of utilizing BC as a phage delivery vehicle for therapeutic purposes. Given that skin infections represent the most prevalent opportunistic infections among patients [27], there is a pressing need for comprehensive research focused on novel antimicrobial strategies targeting these pathogens. The findings presented herein open new avenues for the development of advanced wound dressings incorporating BC as a phage carrier for the treatment of skin infections.

## 2. Results

### 2.1. Carbon Source Selection

**Figure 1:**
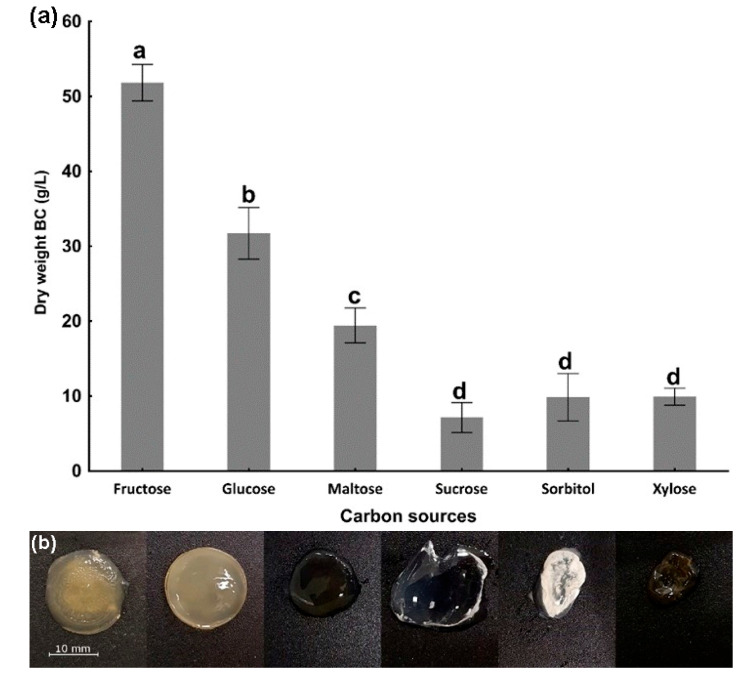
(**a**) Efficiency of BC production (g/L) using various commercial sugars as carbon sources. Data represent means of three replicates, and error bars represent standard deviations. The same lowercase letters above the bars designate homogenous groups at a *p* < 0.05. (**b**) Representative appearance of BC films produced through fermentation using different carbon sources (from the left: fructose, glucose, maltose, sucrose, sorbitol, xylose).

As previously introduced, the choice of carbon source significantly impacts the overall cost of BC production. The volumetric production (g/L) of BC was assessed using modified DSMZ 105 broth, where glucose was replaced with other commercially used sugars. The highest productivity was obtained by using fructose as a carbon source, resulting in the production of 51.80 ± 2.43 g of BC. The lowest BC productivity, in the range of 7.12 ± 2.00 g to 9.93 ± 1.13 g, was obtained with the use of sucrose and xylose, as compared to that achieved with a fructose-based medium (Figure 1a). The obtained cellulose discs differ in both color and transparency. Those produced from fructose, glucose, maltose, and xylose have a yellowish tint and are less transparent (Figure 1b).

### 2.2. Optimization of BC Production

**Figure 2:**
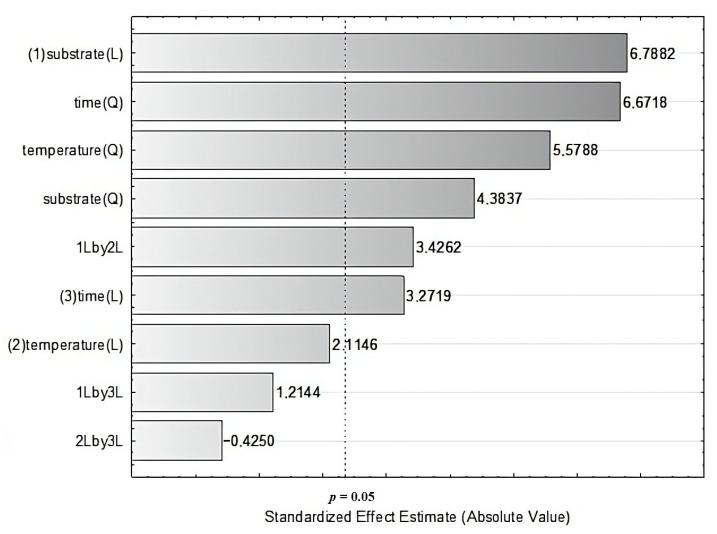
Pareto chart of standardized effects from regression model (linear—L, quadratic—Q and interactive linear—LxL effects).

A Box–Behnken experimental design was employed to assess the simultaneous effects of three independent variables—fructose concentration, temperature, and cultivation time—on BC production. According to the Pareto chart of standardized effects, all three parameters had a statistically significant impact on the measured response (Figure 2, Table 1). Among them, fructose concentration exerted the most pronounced influence, demonstrating both linear and non-linear effects. Similar trends, involving both linear and nonlinear regression coefficients, were also observed for cultivation time. On contrary, the significant impact of cultivation temperature was strictly non-linear. Furthermore, a single linear interaction between fructose content and temperature was confirmed. Coefficients of the polynomial equation were fitted to produce the final regression equation, in which negative quadratic terms render unequivocal maxima in response surfaces (significant terms underlined).

Y = −429.43 − 0.10X_1_ + 28.87X_2_ + 16.89X_3_ − 0.00X_1_X_1_ − 0.58X_2_X_2_ − 0.70X_3_X_3_ + 0.02X_1_X_2_ + 0.01X_1_X_3_ 0.05X_2_X_3_

**Figure 3:**
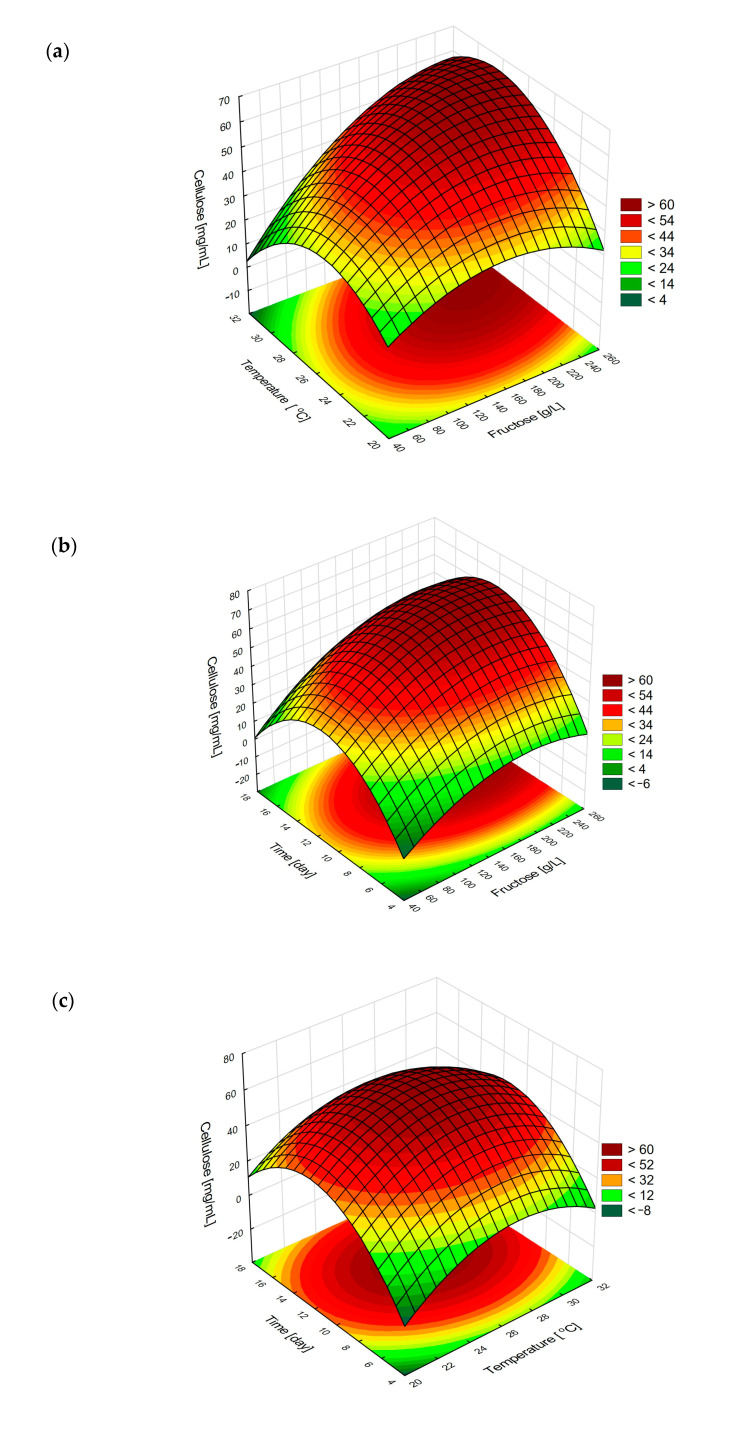
Response surface plots illustrating the combined effects of (**a**) fructose concentration and temperature, (**b**) fructose concentration and cultivation time, and (**c**) cultivation time and temperature on BC production by *K. sucrofermentans*.

Response surface plots illustrate the combined effect of independent variable pairs on the response. The plot in Figure 3a depicts the simultaneous impact of fructose content and cultivation temperature. It confirms that mesophilic temperature range promoted the biosynthesis of BC by the tested strain. Furthermore, elevated fructose content appeared to be beneficial. The asymmetry of the response surface results from the significant interaction between the two independent variables.

The impact of fructose content vs. cultivation time, as shown in Figure 3b, exhibited a clear peak. This suggests that a specific time window must be kept, to maintain highest cellulose yield. The minor asymmetry of the surface was confirmed to have no statistical significance. Non-linearity of the response surface with a convex characteristic is also observed in Figure 3c, which describes the combined effects of cultivation time and temperature.

Hence, optimal values of independent variables could be determined in order to maximize the response, i.e., final content of BC. As inferred from the regression model, the following optimal values for independent variables were determined: fructose content 227.5 g/L, temperature 28.0 °C, and cultivation time 295 h, which correspond to the predicted value of 66.69 g/L cellulose, with a confidence interval 60.25–73.13 g/L. The Box–Behnken model was experimentally validated under these optimized conditions, and the resulting cellulose yield of 63.07 ± 2.91 g/L fell within the predicted confidence interval, confirming both the accuracy and robustness of the model for optimizing BC production.

An analysis of variance (ANOVA) was conducted to assess the significance of both the main and interactive effects of parameters in the developed model (Table 2). Accuracy of the model was further supported by an exceptionally high coefficient of determination (R2 = 0.9953) showing that over 99% of the response variability was explained by the model. In addition, the non-significant “lack of fit” test reinforced the model’s reliability.

### 2.3. BC as a Bacteriophage Carrier

**Figure 4:**
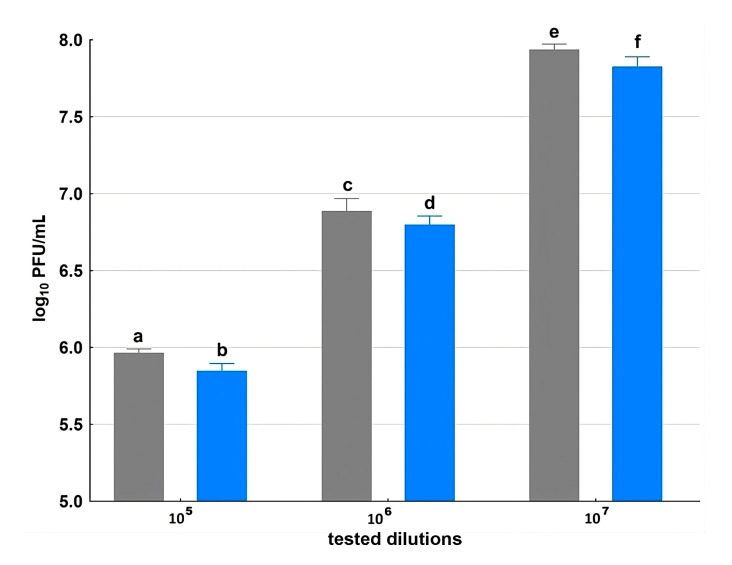
Immobilization of bacteriophage T4 (PFU/mL) on BC. Gray bars indicate the initial titer of phage T4 preparations; blue bars indicate the titer of T4 preparations after incubation with BC discs. Data represent means of three replicates, and error bars represent standard deviations. The same lowercase letters above the bars designate homogenous groups at a *p* < 0.05.

Two phages were employed in this study: *Escherichia coli* bacteriophage T4, one of the best-characterized bacterial viruses and a frequently used model phage, and phage vB_SauS_CS1 (CS1), specific to *S. aureus* and showing high therapeutic potential—particularly in the treatment of wound infections. The comparison of the initial phage titer (PFU/mL) with the concentration of phage particles remaining after incubation with BC disks allowed us to estimate the efficiency of phage adsorption to the carrier. The bacteriophage uptake ability of the BC carrier varied dependent on the phage used. The significant reduction in the T4 phage titer compared to the control demonstrates the successful attachment to the BC (Figure 4). In the range of the initial loading phage concentration from 10^5^ to 10^7^ the immobilization yield shows comparable values. The phage uptake was calculated at 23.5, 19.1 and 22.0% PFU/mL (*p* < 0.05), respectively, along with increasing initial phage concentrations.

**Figure 5:**
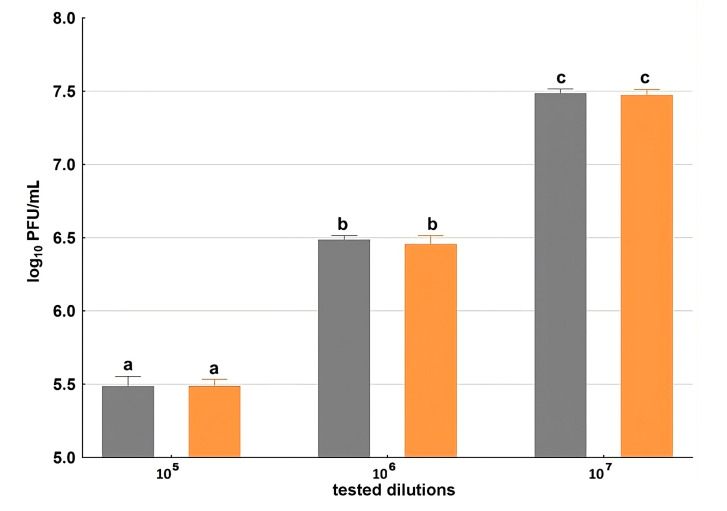
Immobilization of bacteriophage CS1 (PFU/mL) on BC. Gray bars indicate the initial titer of phage CS1 preparations; orange bars indicate the titer of CS1 preparations after incubation with BC discs. Data represent means of three replicates, and error bars represent standard deviations. The same lowercase letters above the bars designate homogenous groups at a *p* < 0.05.

The BC discs showed lower ability to bind the CS1 phage in comparison to T4 (Figure 5). No significant difference was observed between the initial phage titer and the number of phages remaining in solution at each concentration (*p* > 0.05). The phage uptake did not exceed 7%. However, the immobilization technique was found to be advantageous, as it retained a sufficient number of phages on the BC carrier. In addition to the immobilization yield, the zones of bacterial lysis around cellulose disks demonstrate that bacteriophages adhering to the carrier maintained their lytic activity. The diameters of visible zones of bacterial inhibition in the presence of phage-soaked cellulose discs are summarized in Table 3. Both phages T4 and CS1 generated comparable lysis zones, the sizes of which were influenced by the phage concentration.

### 2.4. BC Characterization

**Figure 6:**
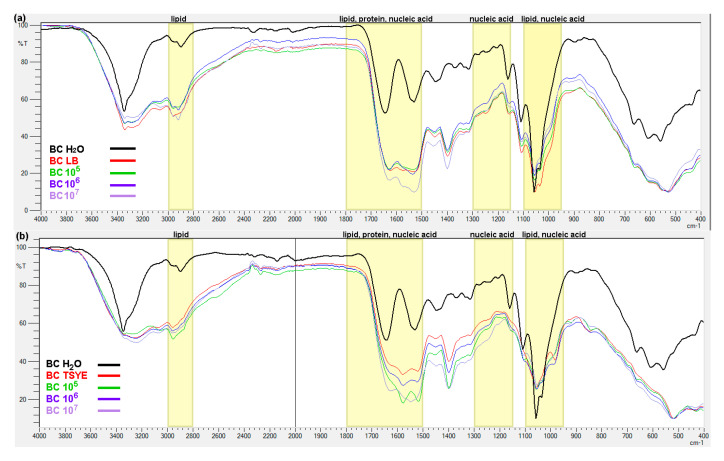
FTIR spectra of BC: (**a**) BC immobilized with bacteriophage T4 and antedated in: water (BC H_2_O), Luria broth (BC LB), sequentially in bacteriophage preparations with titers of 10^5^–10^7^ (BC 10^5^–10^7^); (**b**) BC immobilized with bacteriophage CS1 and antedated in: water (BC H_2_O), Tryptone Soya Yeast Extract medium (BC TSYE), sequentially in bacteriophage preparations with titers of 10^5^–10^7^ (BC 10^5^–10^7^).

Figure 6 presents FTIR spectra of BC synthesized by *K. sucrofermentans*. The spectra exhibit characteristic bands that are indicative of cellulose I, a crystalline form of cellulose typically observed in BC. The control sample demonstrates distinct absorption peaks at 3400, 2900, 1645, 1445, and 1035–1070 cm^−1^, which are commonly attributed to specific molecular vibrations in the cellulose structure [28]. The broad band around 3400 cm^−1^ is attributed to O–H stretching vibrations, reflecting the extensive hydrogen bonding within the BC structure. The absorption band near 2900 cm^−1^ corresponds to the C–H stretching vibrations of CH_2_ and CH_3_ groups present in the glucose units. The band at approximately 1645 cm^−1^ is due to the bending vibrations of adsorbed water molecules, which are typically retained within the highly hydrophilic BC matrix. The peak observed at 1445 cm^−1^ is attributed to symmetric CH_2_ bending vibrations. Finally, the absorption bands in the region of 1035–1070 cm^−1^ correspond to C–O–C stretching vibrations of the glucopyranose ring, which are fundamental components of the cellulose backbone [28].

In the FTIR spectra of both the T4 (Figure 6a) and CS1 (Figure 6b) samples, notable deviations are observed in the spectral regions corresponding to functional groups typically associated with lipids, proteins, and nucleic acids. These differences are most likely due to the varying concentrations of bacteriophage preparations used in the experimental conditions. Specifically, alterations in the peaks related to lipid (around 2900 and 1740 cm^−1^), protein (around 1550 cm^−1^), and nucleic acid (typically observed in the region of 1150–1300 cm^−1^) functional groups suggest that the presence of immobilized bacteriophages could affect the chemical composition of the BC [29].

## 3. Discussion

BC is increasingly being used in various fields due to its beneficial properties. Features like high purity, biocompatibility, excellent mechanical strength, and water holding capacity make it suitable for biomedical applications, such as wound dressings and tissue engineering, as well as in the food and cosmetic industries [30].

The first stage of this study involved the selection of the carbon source for production of BC by *K. sucrofermentans*. It is noteworthy that the appearance of the resulting cellulose discs differed from one another in both color and transparency. The differences in the color of BC can be attributed to pigments formed during the preparation of the media, likely as a result of the Maillard reaction. These colored compounds may have bound to the cellulose fibers, causing their discoloration [31]. Furthermore, the differences in transparency can be explained by variations in the structure of the resulting BC. BC produced from glucose (a hexose) has a high degree of crystallinity (~88%), while BC derived from pentoses and their polyols (e.g., arabitol, xylose) exhibits lower crystallinity (~75%) [32]. X-ray diffraction (XRD) analysis from a study on *Komagataeibacter* sp. W1 shows that media containing various sugars (fructose, glucose, glycerol, mannitol) produce characteristic sharp crystalline peaks, whereas sugars such as lactose and sucrose result in weaker peaks, indicating reduced crystallinity and a more ‘loose’ fiber structure, which confirms our observations [33]. The efficiency of BC production is influenced by various factors. In this study, we specifically focused on temperature, fructose concentration, and cultivation time. The results obtained demonstrate a 27.8% increase in BC yield when a fructose-based broth (DSMZ 105) was used as the carbon source, with cultivation carried out for 7 days at 28 °C.

There is a limited number of studies addressing the combined impact of the same variables investigated in the present work. Nevertheless, our findings are consistent with those reported by Basu et al. [34], who identified sucrose as the optimal carbon source for *Gluconacetobacter hansenii* ATCC 53582. They achieved a maximum BC yield of approximately 40 g/L, which is lower than the yield of around 60 g/L obtained in our study. Similarly, Khiabani et al. [35] reported a productivity of 45 g/L under stirred fermentation conditions (pH = 6, 30 °C, 7 days, 120 rpm) using nabat waste (a mixture containing glucose, fructose, and sucrose) as the carbon source. Consistent with our findings, the optimal cultivation time in their study was also determined to be 7 days.

To reduce the number of experimental runs, we implemented the Box–Behnken design to optimize BC productivity. The utility of Box–Behnken-based modelling was also described by Bae et al. [36], who reported a 57% improvement in BC yield. Their experiments conducted with *Acetobacter xylinum* BPR2001 investigated the effects of fructose, corn steep liquor, dissolved oxygen, and agar concentration. While the carbon source plays a key role in BC production, strain selection is equally critical. For example, Mikkelsen et al. [37] found that mannitol was the most effective carbon source for *Gluconacetobacter xylinus* ATCC 53524, although glucose and fructose also supported relatively high levels of cellulose production. Tabaii and Emtiazi [38] reported glycerol as the optimal carbon source for three strains of *G. xylinus* and two other isolates identified as *Gluconacetobacter*. Moreover, for *A. xylinum*, the optimal cultivation time for BC production was reported as 9 days [39].

While this study primarily focused on optimizing basic cultivation parameters, numerous other strategies have been reported to enhance BC production. Adamopoulou et al. [40] achieved a BC yield of 19.4 g/L by supplementing industrial raisin side-stream extract with thiamin, ascorbic acid, and gallic acid. Amason et al. [41] demonstrated that the addition of gelatin as a template-forming additive resulted in nearly a fourfold increase in BC yield, while also improving the mechanical properties and water-holding capacity of the material. It is worth mentioning that supplementation of the culture medium with vegetable oil also significantly enhanced BC productivity; a 1% oil addition resulted in a 553% increase in the dry weight of BC compared to the control [42].

Data on the application of BC as a carrier for bacteriophages remain limited. Previous studies have investigated BC as a potential biomaterial for cell delivery [25] enzyme immobilization [43], incorporation of probiotic bacteria [19] or bioactive compounds targeting antibiotic-resistant bacteria [44] among others. In this study, we demonstrated that BC has potential as a carrier for phage delivery, particularly for the treatment of skin and wound infections. Fourier-transform infrared (FTIR) analysis was employed to confirm the binding of phages to BC. The minor spectral differences observed may be attributed to the cellulose-to-phage ratio, where the signal from the phages may have been masked by the dominant spectral signature of the BC. These findings are consistent with those of Fuller et al. [29], who reported detectable spectral changes only at bacterial contamination levels of 0.1%.

FTIR analysis revealed only minor alterations in the spectrum following phage immobilization, suggesting that the chemical interactions involved might be too subtle to be thoroughly identified using the applied techniques. To investigate the phenomenon of bacteriophage binding to the BC matrix in greater detail, a range of complementary analytical methods can be employed. For instance, scanning electron microscopy (SEM) and transmission electron microscopy (TEM) enable direct visualization of phage particles on the BC surface, providing morphological evidence of successful immobilization. Similarly, atomic force microscopy (AFM) offers nanoscale topographical information, which can reveal subtle changes in surface roughness or the presence of new nanostructures resulting from phage attachment [45]. To assess the chemical composition of the surface, X-ray photoelectron from phage attachment spectroscopy (XPS) can be used. This technique enables the detection of elements associated with phage capsid proteins and thus supports confirmation of immobilization at the molecular level [46]. Furthermore, fluorescent labeling of phages with dye-conjugated antibodies, combined with confocal microscopy, facilitates the spatial visualization of phage distribution and localization within or on the BC matrix [47]. In addition, enzyme-linked immunosorbent assay (ELISA) employing phage-specific antibodies provides a highly specific and sensitive means to detect and quantify phages immobilized on the BC surface. Together, these techniques offer a comprehensive approach for elucidating the nature and extent of phage–matrix interactions [45].

Nevertheless, the antibacterial activity of phages immobilized within BC was confirmed through experiments in which BC hydrogel discs containing phages were placed on bacterial lawns. The observed zones of bacterial lysis indicate that BC can effectively deliver phages to the site of infection and that the immobilized phages retain their antibacterial efficacy.

The mechanism by which bacteriophages interact with BC remains unclear; however, two possible pathways can be proposed. The first involves passive adsorption of the bacteriophages onto BC via protein–ligand interactions. The second may resemble a “sponge-like” mechanism, in which BC’s exceptional water-holding capacity traps phages within the water retained in the cellulose network. Notably, there are strategies to improve phage binding to BC fibers. Anany et al. [48] found that the durability of bacteriophage adhesion to cellulose membranes could be enhanced by modifying the cellulose surface with positively charged polymers. They used a polyvinylamine polymer dissolved in NaCl to create a positively charged cellulose membrane, which improved bacteriophage binding. Similarly, Vonasek et al. [45] demonstrated that electrostatic interactions could achieve approximately 15–25% phage loading, normalized to the initial titer of the phage solution. Moreover Li et al. [49] reported that genetically modified phage T4, incorporating carbohydrate-binding modules (CBM-T4), could specifically bind to cellulose films, while retaining infectivity after attachment. Moreover Tolba et al. [50] used bacteriophage T4 modified with BCCP and CBM-GFP coding sequences to create immobilized microcrystalline cellulose beads for use in biosensor applications.

Undoubtedly, the use of bacteriophages immobilized on BC in wound therapy creates opportunities for developing dressings with high specificity of action, simultaneously accelerating the healing process and reducing the risk of recurrent infections [51]. In perspective, the findings from this study open several avenues for future research in the field of BC production and its applications. While key factors such as temperature, carbon source concentration, and cultivation time have been optimized to enhance BC yield, further improvements could be achieved by exploring alternative carbon sources and growth conditions. Previous studies have demonstrated that the use of industrial byproducts or alternative carbon sources, such as agricultural waste, can improve BC production and reduce costs [52]. Moreover, the optimization of culture conditions, including the addition of nutrients or growth stimulants, has been shown to further enhance BC yields and its mechanical properties [53].

Importantly, BC’s potential as a carrier for bacteriophages offers promising applications in antimicrobial therapies, especially for wound treatment. Future studies should focus on elucidating the mechanisms underlying BC-bacteriophage interactions, particularly how surface modifications or additives can enhance phage attachment and stability. Understanding these interactions will be crucial for developing effective BC-based delivery systems for bacteriophages in clinical settings.

## 4. Materials and Methods

### 4.1. Materials

Bacteriophages CS1 (DSMZ 105264), T4 (DSMZ 4505) and bacterial strains *Komagataeibacter sucrofermentans* DSM 15973 (formerly *Acetobacter xylinus* subsp. *sucrofermentans*, Gluconacetobacter subsp. *sucrofermentans*), *Escherichia coli* (DSM 613), and *Staphylococcus aureus* (DSM 105272) were obtained from the Leibniz Institute DSMZ—German Collection of Microorganisms and Cell Cultures GmbH (Braunschweig, Germany). The following reagents and culture media were used: Luria Broth (A&A Biotechnology, Gdańsk, Poland), fructose, CaCO_3_ (Chempur, Piekary Śląskie, Poland), glucose, agar (Merck, Darmstadt, Germany), maltose, sucrose, sorbitol, and xylose (Sigma-Aldrich, St. Louis, MO, USA).

### 4.2. Methods

#### 4.2.1. Carbon Source Selection

*K. sucrofermentans* was used to produce BC. The bacteria were cultivated in modified DSMZ 105 broth, which contained 10.0 g/L yeast extract, 20.0 g/L CaCO_3_, and 100 g/L of specific carbon sources: fructose, glucose, maltose, sucrose, sorbitol, and xylose. A total of 2.0 mL of the broth containing different carbon sources was transferred to a 24-well plate and inoculated with 15 µL of a 48-h bacterial preculture. Static cultivation was carried out at 28 °C for 7 d. After the cultivation period, BC was removed from the wells, briefly dried on Whatman paper for 5 s, weighed, lyophilized, and then re-weighed to obtain the dry mass. The results were expressed as the dry mass of BC produced per 1 L of medium. The experiment was performed in triplicate.

#### 4.2.2. Optimization of BC Production

The production of BC in culture of *K. sucrofermentans* was optimized using the Box–Behnken experimental design. A non-linear multiple regression model was built to establish the simultaneous influence of three predictors, fructose concentration (X_1_), temperature (X_2_), and cultivation time (X_3_), on the response (cellulose yield). Each predictor was applied at three experimental levels (−1; 0; +1), as listed in Table 4.

The experimental layout comprised 20 runs with different combinations of variable levels (Table 5), while the central point was executed in eight replications. Coefficients of the following regression equation were determined:

Y = β_0_ + β_1_X_1_ + β_2_X_2_ + β_3_X_3_ + β_11_X_1_X_1_ + β_22_X_2_X_2_ + β_33_X_3_X_3_ + β_12_X_1_X_2_ + β_13_X_1_X_3_ + β_23_X_2_X_3_

where Y—the predicted response, X_1_, X_2_, X_3_—independent variables (fructose content, temperature, cultivation time, respectively), β_0_—the intercept, β_1_, β_2_, β_3_—linear regression coefficients, β_11_, β_22_, β_33_—square regression coefficients, β_12_, β_13_, β_23_ interaction regression coefficients.

#### 4.2.3. BC Disks Preparation

Based on the obtained results, BC discs were prepared under optimized conditions: 230.0 g/L fructose, 12 d of static cultivation at 28 °C in a 24-well plate with 2 mL of medium. The purification process involved treatment with 0.1 M NaOH solution at 80 °C for 2 h, followed by washing in distilled water for 1 h. The purified BC discs were then transferred to fresh distilled water and sterilized for further analysis.

#### 4.2.4. BC as a Bacteriophage Carrier

The phage preparations were prepared using two-stage culture method [54]. Three consecutive decimal dilutions of the bacteriophage preparation were prepared, resulting in concentrations of 4.5 × 10^5^ PFU/mL, 4.5 × 10^6^ PFU/mL, and 4.5 × 10^7^ PFU/mL. Each dilution was transferred into four tubes to a volume of 20 mL each. One tube served as a control of phage titer. Four BC discs were placed into the other three tubes. All tubes were incubated for 1 h at 37 °C and 100 rpm. After incubation, three BC discs were removed and placed on the petri dishes with a 24-h bacterial lawn. The petri dishes were then incubated for 24 h at 37 °C. The fourth BC disc was frozen for FTIR analysis. The size of the lysis zones was measured in four dimensions using a caliper. Phage titer in the control and in the suspension remaining in each tube was determined by the double layer agar plate method [55].

#### 4.2.5. BC Characterization

The identification of functional groups was performed using an IRSpirit Fourier Transform Infrared (FTIR) spectrophotometer (Shimadzu, Kyoto, Japan). The BC production yield was determined using a Triad freeze dryer (Labconco, Kansas City, MO, USA) and an analytical balance (Radwag, Radom, Poland).

#### 4.2.6. Statistics

Data from the experiments on carbon source selection and the use of bacterial cellulose (BC) as a bacteriophage carrier are expressed as means ± standard deviations. Statistical analysis was performed using STATISTICA 14 software (TIBCO Software Inc., Palo Alto, CA, USA), including analysis of variance (ANOVA) followed by Tukey’s post-hoc test, with a significance level set at *p* < 0.05. Additionally, response surface methodology (RSM) based on a Box–Behnken design was employed to model and optimize experimental conditions, enabling the design of experiments, regression analysis, generation of response surface plots, and response optimization.

## 5. Conclusions

BC has a wide range of applications, including as a biomaterial carrier for biologically active molecules and compounds. Its production is influenced by various factors. In this study, we optimized BC production concerning fructose concentration, temperature and time of cultivation. Under the optimized conditions—fructose concentration of 227.5 g/L, temperature of 28 °C, and cultivation time of 295 h—we achieved a BC yield of 66.69 g/L, representing a 27.8% increase compared to non-optimized conditions. Furthermore, the ability of bacteriophages to bind to BC was demonstrated. Although the mechanisms underlying phage immobilization on bacterial cellulose are not yet fully elucidated, the results indicate that BC holds potential as a carrier for bacteriophages. To the best of our knowledge, this study is the first to show that BC can feasibly be used as a delivery system for bacteriophages in therapeutic applications. The results of this research pave the way for creating advanced wound dressings that use BC as a phage carrier to help treat skin infections.

## Figures and Tables

**Table 1:** Summary of the regression model effects.

Variable	Coefficient	Standard Error	t-Value	*p*-Value
Intercept	30.7508	1.6164	19.0237	0.0000
X_1_	26.8775	3.9595	6.7882	0.0003
X_2_	11.4806	2.6189	4.3837	0.0032
X_3_	8.3725	3.9595	2.1146	0.0723
X_1_X_1_	14.6106	2.6189	5.5788	0.0008
X_2_X_2_	12.9550	3.9595	3.2719	0.0136
X_3_X_3_	17.4731	2.6189	6.6718	0.0003
X_1_X_2_	19.1850	5.5995	3.4262	0.0110
X_1_X_3_	6.8000	5.5995	1.2144	0.2634
X_2_X_3_	−2.3800	5.5995	−0.4250	0.6836

**Table 2:** Analysis of variance (ANOVA) of the regression model, including combined linear and quadratic effects (L + Q) of input variables and interaction terms (X_a_ × X_b_), where SS—sum of squares, MS—mean square, df—degrees of freedom.

Regression Model Component		SS	df	MS	F-Ratio	*p*-Value
X_1_: fructose [g/L] L + Q		2047.34	2	1023.67	32.65	0.0003
X_2_: temperature [°C] L + Q		1116.06	2	558.03	17.80	0.0018
X_3_: cultivation time [day] L + Q		1731.37	2	865.68	27.61	0.0005
interactions	X_1_ × X_2_	368.06	1	368.06	11.74	0.0110
	X_1_ × X_3_	46.24	1	46.24	1.47	0.2640
	X_2_ × X_3_	5.66	1	5.66	0.18	0.6836
Lack of fit		83.04	3	27.68	0.88	0.4948
Residual error		219.48	7	31.36		
Total SS		6763.78	19			

R2 = 0.9553, adjusted R2 = 0.9150.

**Table 3:** Mean diameter of inhibition zones of bacteria with the addition of cellulose disks soaked with bacteriophages at different titer (PFU/mL) compared to the control cellulose disks diameter. The same superscript letters designate homogenous groups within the same column at *p* < 0.05.

Bacteriophage	CS1	T4
Tested dilution (PFU/mL)	Disk diameter (mm)	
Control	16.67 ± 0.52 ^a^	16.44 ± 0.50 ^a^
10^5^	19.01 ± 0.76 ^b^	18.67 ± 0.43 ^b^
10^6^	19.63 ± 1.04 ^c^	19.59 ± 1.02 ^c^
10^7^	20.12 ± 0.58 ^d^	19.57 ± 0.85 ^c^

**Table 4:** Experimental values of independent variables.

	Unit	−1	0	+1
X_1_: fructose	g/L	50	150	250
X_2_: temperature	°C	21	26	31
X_3_: cultivation time	day	6	11	16

**Table 5:** Experimental layout of the Box–Behnken design, with the obtained response outcomes and predicted response values.

Independent Variable				Cellulose g/L	
Run	X1	X2	X3	Actual Response	Predicted Response
1	0	1	−1	26.09	26.61
2	0	−1	−1	11.36	15.86
3	−1	0	−1	16.46	14.32
4	1	0	−1	37.28	34.40
5	−1	−1	0	28.03	25.67
6	1	−1	0	34.98	33.36
7	−1	1	0	13.24	14.86
8	1	1	0	58.56	60.92
9	−1	0	1	17.60	20.48
10	1	0	1	52.02	54.16
11	0	−1	1	31.71	31.19
12	0	1	1	41.68	37.18
13	0	0	0	60.30	59.79
14	0	0	0	53.33	59.79
15	0	0	0	58.13	59.79
16	0	0	0	53.15	59.79
17	0	0	0	55.70	59.79
18	0	0	0	65.08	59.79
19	0	0	0	66.63	59.79
20	0	0	0	66.05	59.79

## Data Availability

The datasets generated during and/or analyzed during the current study are available from the corresponding author upon reasonable request.

## Abbreviations

The following abbreviations are used in this manuscript:

BC	Bacterial cellulose
Phages	Bacteriophages
CS1	*Staphylococcus aureus* phage vB_SauS_CS1
